# Women's Uncongenial Occupations

**Published:** 1890-04-05

**Authors:** 


### The Doctor's Pulpit.

### WOMEN'S UNCONGENIAL OCCUPATIONS.

Many men, perhaps the majority, would prefer to see women, as a class, entirely free from the necessity of following any definite occupation at all. Mr. Walter Besant, if we mistake not, would furnish every woman with a liberal competency, and surround her with friends, flowers, amusements, cloudless skies, and sunshine all through the year. Yet Mr. Besant is a middle-aged man, and appears to know the full and eternal significance of the word "duty." The lover under thirty years of age, enwrapt in the bliss of his first *grande passion*, may properly indulge the sentiments we have given Mr. Besant credit for. More than this: he is not worthy of the name of lover at all unless he longs to surround the woman of his worship with an infinite atmosphere of unalloyed beatitude. But first love is a temporary condition: not by any means a delusion, however. It is so dominant in its intensity that it excludes many other things which go to make up both life and the happiness of life. When reason at length gains the ear of the lover it does not necessarily make his love either feebler in intensity or less enduring. But it makes it different: and the difference is favourable to a truer and juster view of what constitutes worthy and perfect life.

Women, or rather girls just dawning into womanhood, seem to be in their native element in a kind of universal flower garden. And because they seem so entirely at home there, so happy, and so natural, men do them the double injustice of desiring to keep them always there, and of thinking that they themselves never feel any impulsions towards nobler and more rational ways of living. But women need occupation as much as men do; and they are quite as conscious of their need. Not only so, but their versatility of mind and their natural impulses incline them to occupations of the nobler and more generous sort. They can, however, adapt themselves to any kind of occupation; and provided they have sufficient health and strength, they may effect as good results as men.

It is quite a common thing to hear people discuss the question of the congeniality or otherwise of their employment. Perhaps women give more attention to the subject than men. It will generally be found, however, that the persons of both sexes who discuss the question seriously are young. Men and women who have reached middle life have mostly made up their minds that whether their work is congenial or not it has to be done. They do it as a matter of business and duty; and regarding it in this light, they think it necessary and best to do it as well as possible. There has for some time been going on in our columns a discussion about the congeniality of nursing as an employment for certain kinds of women. Nurses, as might have been expected, take exactly the same kind of views on the subject as are prevalent in the great world outside hospitals. Some of them think that the true nurse is "born and not made"; and that she experiences a kind of supernatural call to her work, exactly in the same way as the puritan divine or the old-fashioned king used to do. Others again think that it is open to any woman, whether she has special aptitudes or not, to choose the profession of nursing as a means of making an honest livelihood. The latter class sometimes find the work of the sick room decidedly uncongenial, not to say repulsive. The former are inclined to retort upon them that they should not be in the nursing profession at all; and that in fact no woman is fit for the peculiar duties of the sick room except the "born nurse."

What do common sense and experience say on this important question? They say that in considering a subject of the kind it is desirable to take it out of its local and temporary surroundings and to place it side by side with other similar subjects. More light and guidance are likely to be discovered by discussing the general question of "uncongenial employments" than by considering the subject of the congeniality or otherwise of "nursing," by itself. The clerical profession, the medical, the legal, the teaching, the coat-making, the sugar-selling, and the chimney-sweeping, all stand in much the same relation, of congeniality or uncongeniality, to their members, as nursing does to its followers. Ought a doctor to be "born and not made"? Or a lawyer? Or a sweeper of chimneys? Let us answer this question in the affirmative for a moment, and see where the answer will lead us. It will certainly lead us in the first place to such a scarcity of doctors, lawyers, and chimney-sweepers that nobody except the very richest will be able to obtain either medical treatment or legal advice, or even to get his chimneys swept. Not one in fifty of practising doctors could properly be called a "born physician." Yet probably forty-five in every fifty actual medical men do their work satisfactorily and well; and find it by habit reasonably congenial. The same may be said of clergymen, lawyers, coat-makers, sugar-sellers, chimney-sweepers, and indeed of every other professional class that has an occupation separate and distinct from those of its fellow men. The truth is that when we say a man is "born" to a thing, we merely mean that he is born with unusual ability and capacity: in short, that he is a genius. Now geniuses in this sense bear a very small proportion to the whole of the members of any particular calling, and to mankind in general.

The practical question for a nurse, as for any other woman to ask, is not, does she find her occupation entirely congenial, but does she believe in duty: and is she minded to practise it? A good will can make repulsive duties pleasant, and a discontented mind can make the most congenial work hateful. Women, much more than men, are dependent on their states of mind. A man with his stolid and unemotional disposition can keep doggedly at work in which he has not the slightest interest or pleasure, for a life-time. But a woman, who has no interest in her occupation, flags and becomes listless and melancholy. "Exactly so," say the pleaders for "born" nurses, and "that is the very reason why no woman should take up nursing who has not a special love for it." But, as we have pointed out, the universal application of that rule would give us about one nurse where we have, and need, fifty now. It is clearly, therefore, impracticable. If we want a principle which shall be applicable to all present and potential nurses it must be much broader than the "special aptitude" and "special calling" rule. It is not necessary, as it is not possible, that every woman desirous of following the calling of a sick nurse should have a special and particular love for sick nursing; but it is necessary that every such woman shall have a special and particular love for "duty." For our part we are convinced that if a thousand women who love duty for duty's sake be set to sick nursing, and a thousand other women who think they love nursing for nursing's sake be employed in it, there will be found a far larger proportion of capable nurses among the "duty" lovers than among the lovers of "nursing" exclusively. The same rule applies to every other occupation followed by women. The congeniality or uncongeniality of work may be a matter of great importance to the flighty and the feeble minded whose conscience is weak and whose selfishness is strong. But to the true woman who understands the real significance of life, who believes in God, who knows to some extent what Jesus Christ was and is to the world—to this woman uncongeniality of work is a very unfamiliar word. Is the work there? Does it demand to be done? Is it plain and evident that she can and ought to do it? Then she does it, valiantly, thoroughly, and with happiness to herself. Whether it be the soothing of a fractious child, the caring for the helpless and the old, or the performance of the most repulsive offices for the sick—what is it to her? It is her divinely appointed duty; and in it she finds the deepest satisfaction of her heart. Let no woman therefore turn away from sick nursing or any other calling on the sole ground of its uncongeniality. If it is plain that present duty points to this as the present task of life, the only right course is to keep steadfastly and resolutely to it.

